# Investigating preschool-aged chronotype and social jetlag as predictors of early adolescent diet and BMI *z*-score: an eight-year follow-up from the DAGIS study

**DOI:** 10.1038/s41366-024-01702-4

**Published:** 2024-12-19

**Authors:** Anna M. Abdollahi, Ilona Merikanto, Henna Vepsäläinen, Xinyue Li, Emmi Tilli, Henna Peltonen, Ilse Tillman, Carola Ray, Josefine Björkqvist, Eva Roos, Reetta Lehto, Maijaliisa Erkkola

**Affiliations:** 1 Department of Food and Nutrition, University of Helsinki, Helsinki, Finland; 2 Faculty of Medicine, University of Helsinki, Helsinki, Finland; 3 Department of Public Health and Welfare, Finnish Institute for Health and Welfare, Helsinki, Finland; 4 Orton Orthopaedics Hospital, Helsinki, Finland; 5 School of Data Science, City University of Hong Kong, Hong Kong SAR, China; 6 Folkhälsan Research Center, Helsinki, Finland; 7 Department of Food Studies, Nutrition and Dietetics, Uppsala University, Uppsala, Sweden; 8 Department of Public Health, University of Helsinki, Helsinki, Finland

**Keywords:** Risk factors, Paediatrics

## Abstract

**Background/Objectives:**

Circadian health plays an important role in overall well-being. The objective of this study was to examine whether potential indicators of circadian disruption, such as exhibiting a later chronotype or greater social jetlag, in preschool-age could predict dietary habits or BMI *z*-scores in an eight-year follow-up.

**Subjects/Methods:**

Our data included 210 children who participated in the DAGIS Survey in 2015–2016 (baseline, mean [SD] age: 4.69 [0.89] years) and DAGIS Next in 2023 (follow-up, age: 12.03 [0.90] years). Chronotype and social jetlag were calculated from baseline sleep measures assessed from 7-day actigraphy. Diet was assessed at follow-up with a Food Frequency Questionnaire, which evaluated the weekly consumption frequency of (1) fruits and vegetables and (2) sugary foods and drinks. BMI *z*-score based on Finnish growth references was calculated from height and weight measures from baseline and follow-up. Associations were analyzed with linear regression models.

**Results:**

Follow-up BMI *z*-score was predicted by both preschool-aged chronotype (β-est: 0.22 [95% CI: 0.01, 0.42] *p* = 0.03) and social jetlag (β-est: 0.33 [95% CI: 0.02, 0.65], *p* = 0.04) after covariate adjustment. No associations were observed between preschool-aged chronotype or social jetlag and preadolescent fruit and vegetable or sugary food and drink consumption.

**Conclusions:**

Having a later chronotype and greater social jetlag during preschool age predicted a higher preadolescent weight outcome. Obesity prevention initiatives should include efforts to reduce the risk of circadian disruption among young children, by accounting for chronotype and aiming to reduce social jetlag in interventions.

## Introduction

Identifying the determinants of childhood overweight and obesity is fundamental to decreasing the prevalence and metabolic risks of obesity later in life [1, 2]. While global obesity rates have only moderately increased in 2–4-year-old children, the prevalence of older children (5–19 years old) and adults with overweight and obesity has rapidly risen in recent decades [3, 4]. The main behavioral factors influencing overweight and obesity are diet, sedentary behavior, physical activity, and sleep, of which the latter has only recently received more recognition in regard to obesity prevention [5, 6].

Among sleep-related factors, chronic circadian misalignment (i.e., the consistent discrepancy between biological and behavioral sleep-wake rhythms) is identified as a potential risk factor for obesity in adolescents and adults [7–9]. Risk for circadian disruption can be indirectly measured with sleep regularity markers, such as variability in sleep schedules (i.e., during shift work), later chronotype (i.e., via innate circadian typology misaligned with societal schedules), or greater social jetlag (i.e., the difference between sleep schedules on work/school days and days with unconstrained wake-up times). In addition to obesity, these risk factors of circadian disruption have been associated with type 2 diabetes, cardiovascular disease, and metabolic syndrome among adults [10–15]. The mechanisms remain unclear, but controlled laboratory studies that induce acute circadian disruption by altering the timing of sleep windows have demonstrated impaired glucose metabolism in states of misalignment [16], even independent of sleep duration [17]. A later chronotype and greater social jetlag in school-aged children have been associated with less healthy diets, hedonistic eating behaviors, and higher energy intake [18–22]. Undoubtedly, the negative effects of circadian disruption can be observed from pre-adolescences to adulthood. However, it remains unclear how early the potential effects of circadian misalignment, including increased risk for obesity, begin to manifest. Since overweight and obesity, as well as the accompanying metabolic risks, often carry over from childhood into adulthood, it is crucial to recognize pivotal life stages and begin prevention efforts as early as possible [1, 2].

In general, young children grow at a rapid pace, require more sleep, and exhibit an earlier chronotype [23, 24]. Pubertal changes delay circadian rhythms such as sleep-wake rhythm, which, alongside increased autonomy and social changes, contribute to adolescents often being characterized by a later chronotype [25, 26]. Since there are larger disparities between innate circadian typology and societal schedules (i.e., school start time) during adolescence, it is often considered a stage in life where misalignment tends to be the greatest [23, 25]. Although chronotype may shift slightly with age, environment, and behavior, it is underlined by a strong, stable genetic basis [27, 28]. Though young children typically exhibit earlier chronotypes, late chronotype preferences have been consistently identified among preschool-aged children as well [29–31], though the associations with weight and diet outcomes remain largely understudied.

In our previous study among preschoolers, having a later chronotype was associated with lower vegetable and higher sugary food consumption [32]. However, no associations were observed between chronotype and energy intake, or between social jetlag and any dietary factors. Studies with preschool-aged children at risk for obesity have observed greater social jetlag associated with increased energy intake and diet patterns higher in energy-dense foods [33, 34]. Despite this, there is a shortage of studies exploring risk factors for circadian misalignment, such as later chronotype or greater social jetlag, in association with weight outcomes among preschool-aged children [35]. Moreover, previous findings revealed a null association between social jetlag and weight status among preschoolers [36], which is inconsistent with results from older populations [6–9]. Therefore, circadian disruption in preschool age (i.e., when scheduled weekday routines generally begin) may initiate the risk for an unhealthy diet or altered metabolism and, over time, increase the risk for obesity.

The potential lagged effects of chronic circadian misalignment may contribute to understanding why overweight and obesity appear to be more prevalent among school-aged children than preschoolers. Follow-up studies examining the association of either chronotype or social jetlag with diet and weight outcomes are scarce and, to our knowledge, none have a follow-up period extending from preschool age to early adolescence. This eight-year follow-up study is the first to investigate the associations of preschool-aged chronotype and social jetlag with early adolescent diet and weight outcomes. We anticipated that a later chronotype and greater social jetlag during preschool age would predict less healthy dietary habits in early adolescents, including less frequent consumption of fruits and vegetables and more foods and drinks with high added sugar content. Furthermore, we hypothesize that children with an increased risk of circadian misalignment during preschool years, as indicated by later chronotype and greater social jetlag, may be at risk for increased BMI z-score during early adolescence.

## Methods

### Study population

The study population consisted of preschool-aged participants from the DAGIS (Increased Health and Wellbeing in Preschoolers) Survey conducted between September 2015 and April 2016 in Southern and Western Finland. With parent(s) or guardian informed consent, 864 children and their families (24% of those invited) participated in the DAGIS Survey (hereafter referred to as the baseline), which aimed to investigate the energy-balance-related behaviors among preschool-aged children in Finland [37]. The flow chart detailing the baseline study participation has been published elsewhere [37].

**Fig. 1:**
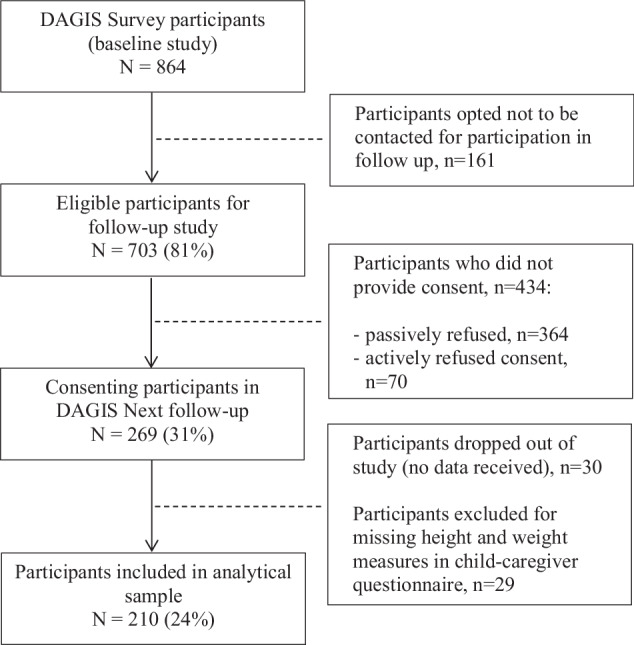
Flow chart of participants in the DAGIS Next follow-up study.

An eight-year follow-up survey, DAGIS Next, was conducted in 2023. All families from the baseline study who consented to future contact and provided information for contact (*n* = 703, 81% of baseline participants) were invited to the follow-up study. Overall, 269 families (31% of baseline participants) provided consent for participation. For the follow-up survey, the parent(s) or guardian provided informed consent and the children provided assent for participation. The analytical data (*n* = 210, 78% of those consented) for this study consisted of children who had participated in the baseline study and had reported anthropometric and food frequency questionnaire (FFQ) data in the follow-up study. Figure 1 illustrates follow-up study participation.

When comparing children who only participated in the baseline study or were excluded due to insufficient follow-up data (*n* = 654) with those included in the analytical sample (*n* = 210), no significant differences were observed in sociodemographic or behavioral variables (*p* > 0.11), except for parental education level (*p* < 0.01, Supplementary Table 1). On average, parents of children excluded from the current study population had a lower education level than those who were included (lowest education levels 27% and 10%, respectively). The University of Helsinki Ethical Review Board in the Humanities and Social and Behavioral Sciences reviewed the baseline study in 2/2015 (#6/2015) and follow up in 1/2023 (#6/2023).

### Weight assessment

Baseline height and weight were measured by trained researchers during preschool visits. Height was measured with stadiometers (SECA 217) to the nearest 0.1 cm. Weight was measured with portable bench scales (CAS PB-100/200) to the nearest 0.01 kg. In the follow-up study, respondents were asked to report the height, weight, and date of measure of the child from their annual school nurse record, which parents and legal guardians can access through Finland’s electronic health record services (My Kanta Pages). In addition, respondents were instructed to measure the height and weight of the child at home. If a nurse’s record was unavailable or the height and weight were not documented within the past 12 months, self-measured reports were utilized (*n* = 81). BMI *z*-scores were calculated based on Finnish growth references [38]. Moreover, sensitivity analysis was conducted using International Obesity Task Force reference values [39].

### Sleep assessment from baseline

During the baseline study, children were instructed to wear a provided ActiGraph wGT3X-BT activity monitor (Pensacola, FL, USA) consecutively for one week, removing the device only for water-based activities. More details on the actigraphy sleep measures and reliability are described elsewhere [36]. In short, a data-driven Hidden Markov Model previously validated against polysomnography was used to identify sleep-wake epochs [40] (R package “hmmacc”), and it has been used for children in several studies [32, 41, 42]. Sleep variables were calculated for children with sleep data for seven nights. Of the analytical sample, 23% (*n* = 49) of children had missing sleep data, of which a third (*n* = 16) did not wear the device during baseline assessment and the rest (*n* = 33) had insufficient data (see Statistical analysis section for details on how missing data was handled). Nights between Friday-Saturday and Saturday-Sunday were defined as weekend nights for sleep assessment.

Chronotype was calculated as a continuous variable from the midpoint of sleep (half the time between sleep onset and wake-up time) from two weekend nights, and followed the formula for corrected mid-sleep time on free days based on the Munich Chronotype Questionnaire [23]. Calculations were based on the assumption that sleep on weekend nights was free from a set schedule since no child attended preschool on weekend days. For children with a mean sleep duration on weekends longer than on weekdays, the weekend midpoint of sleep was adjusted for possible sleep debt accumulated from weekday nights. The adjustment calculation is as follows: mean midpoint of weekend sleep– (mean weekend sleep duration − mean weekly sleep duration) / 2 [23]. A later clock time represents a later chronotype. Social jetlag was calculated as the difference between the weekend and weekday midpoint of sleep. Sleep duration was the period between sleep onset and wake-up times.

### Dietary assessment from follow-up

Children’s food consumption was measured with FFQs during the follow-up study. The FFQ was updated from a 47-item child FFQ with acceptable validity [43] and reliability [44] to a 66-item FFQ that included more plant-based foods and drinks as well as items appropriate for older children. The child was instructed to fill out the questionnaire together with a parent or guardian. The answer options included “never, “once a week”, “2–3 times per week”, “4–5 times per week”, “daily or almost daily (6–7 times per week)”, “2 times per day”, and “3 times per day or more”. The FFQ responses were scaled to numerically reflect weekly consumption frequency (i.e., 0, 1, 2.5, 4.5, 6.5, 14, and 21, respectively).

Based on previous cross-sectional associations found between having a later chronotype and higher sugary food consumption as well as lower vegetable consumption [32], the current study focused on examining consumption of fruits, vegetables, and sugary foods, which also aligns with the main focus of the DAGIS project. The summed FFQ food items used to assess consumption frequencies of fruits, vegetables, and sugary foods and drinks are shown in Table 1.

**Table 1:** Consumption frequency outcome variables derived from food frequency questionnaire items in DAGIS Next study participants (*n* = 210).

Consumption frequency outcome variable (times/week) median (interquartile range)	Consumption frequency sub-grouping (times/week) median (interquartile range)	FFQ items included to assess consumption frequency (times/week) median (interquartile range)
Fruits and vegetables 17.0 (14.5)	Vegetables 9.0 (10.0)	Fresh vegetables 6.5 (7.5)
		Cooked and canned vegetables (excluding potatoes) 1.0 (2.5)
	Fruits and berries 7.0 (5.5)	Fresh, frozen, and canned fruits 4.5 (4.0)
		Fresh or frozen berries 2.5 (1.5)
Sugary foods and beverages 14.5 (9.0)	Sugary everyday foods 5.0 (6.0)	Flavored dairy-based yogurts and quark 2.5 (1.5)
		Dairy-based puddings 0.0 (1.0)
		Flavored plant-based yogurts and quarks 0.0 (0.0)
		Plant-based puddings 0.0 (0.0)
		Sugar-sweetened cereals and muesli 1.0 (2.5)
		Berry and fruit soups 0.0 (1.0)
	Sugary treats 6.5 (4.0)	Sweet pastries, including doughnuts, Danish pastries, cakes, cupcakes, sweet rolls, and pies 1.0 (1.5)
		Sweet biscuits 1.0 (1.0)
		Chocolate 1.0 (1.5)
		Sweets 1.0 (1.5)
		Ice cream, plant-based frozen desserts, sorbet, and popsicles 1.0 (1.5)
	Sugar-sweetened beverages 2.5 (2.5)	Sugar-sweetened soft drinks 1.0 (2.5)
		Sugar-sweetened juice drinks 1.0 (2.5)

### Covariate assessment

Parents reported the child’s sex and date of birth. Age was calculated using the date of birth and the date of anthropometric measures, as recorded by the researcher for baseline and reported by the participant for follow-up. Analyses were adjusted for baseline age and the number of follow-up years as continuous variables. As a proxy for socioeconomic status, the highest reported household parental education level from baseline data was further categorized as low (i.e., high school or vocational diploma or less), medium (associate or bachelor’s degree), and high (i.e., master’s, licentiate or doctoral degree). As an additional descriptive variable, parents reported how many adults lived in the household. This was then converted into a dichotomous variable reflecting whether the household was a single-parent family or not.

### Statistical analysis

Baseline and follow-up characteristics were compared with McNemar’s chi-squared test for categorical variables and paired *t*-tests for continuous variables. Multivariable linear regression was used to assess associations between predictor (chronotype, social jetlag, and sleep duration) and outcome (diet and weight) variables. Since dietary variables were skewed, they were square-root transformed for regression analyses. The potential confounding variables in regression models were selected based on knowledge of variables that have been examined in previous research, including age, sex, socioeconomic status, and sleep duration [36, 45]. Regarding regression analyses, collinearity of independent variables and model assumptions were assessed. Model 1 had no adjustments, except for baseline BMI *z*-score in models where follow-up BMI *z*-score was the outcome. Model 2 additionally adjusted for child’s baseline age, sex, sleep duration, parental education level, and time to follow-up. To gain additional insight into the development of obesity, cross-sectional associations of chronotype and social jetlag with BMI-*z*-score were examined from baseline measures. Tests for effect modification performed by the inclusion of interaction terms between sleep traits and sex were not significant and, therefore, all subjected were analyzed together.

As a sensitivity analysis, all participants with follow-up height and weight measured at home were excluded from the study population (*n* = 81). Analysis was also conducted with more detailed food sub-groupings presented in Table 1, i.e., vegetables, fruits and berries, sugary everyday foods, sugary treats, and sugar-sweetened beverages. However, since the results remained unchanged (data not shown), combined food grouping results are reported. Additionally, sleep-related variables with significant associations were further divided into tertiles to facilitate the interpretation of the findings.

Within the regression models, missing independent variables were handled with multiple imputation using the multivariate imputation by chained equation (MICE) algorithm [46]. Since 76% of the observations were complete, 25 imputations were performed with 10 iterations [47]. Further details of missing observations in the independent variables are presented in Supplementary Table 2. Fully adjusted analyses excluding imputations yielded similar results and are presented in Supplementary Table 3. To avoid using imputed values on outcome variables, the additional multivariable linear regression analysis also assessing sleep variables with change in BMI z-score (baseline BMI *z*-score subtracted from follow-up BMI *z*-score) and baseline BMI *z*-score were assessed without multiple imputation. Analysis were performed with R (version 4.3.1), with two-sided tests and a significance level of *p* < 0.05.

## Results

### Study population

Baseline and follow-up socio-demographic and behavioral variables of the study population are presented in Table 2. Of the total 210 children (48% girls), the mean (SD) follow-up period for the children was 7.35 (0.31) years. Mean change in BMI *z*-score between baseline and follow-up was 0.09 (0.94). There were no significant pairwise differences between children’s baseline and follow-up BMI *z*-score (*p* = 0.09) or categorized weight status (*p* = 0.27). Children in the analytical sample with any missing data (*n* = 51, 24%) did not differ in by any socio-demographic or behavioral variables compared to those without any missing data.

**Table 2:** Descriptive table of baseline and follow-up data from the DAGIS^a^ study participants (*n* = 210).

	Baseline		Follow-up	
	mean (SD) or *n* (%)	minimum–maximum	mean (SD) or *n* (%)	minimum–maximum
Age (years)	4.69 (0.89)	2.77 to 7.09	12.03 (0.90)	9.91 to 14.29
Sex (female)	100 (48%)			
Single parent household	12 (6%)			
Parental education level^b^				
Low	21 (10%)			
Medium	82 (39%)			
High	106 (51%)			
Weight-related variables^c^				
Overweight and obesity	32 (15%)		41 (20%)	
BMI *z*-score	−0.05 (0.97)	−2.95 to 2.05	0.03 (0.96)	−2.70 to 2.61
Sleep variables				
Chronotype (hh:mm)	02:16 (00:39)	01:00 to 03:59		
Social jetlag (min)	25 (26)	−42 to 94		
Sleep duration (hours)	9.73 (0.48)	8.50 to 11.24		
Diet variables (times/week)				
Fruits and vegetables^d^			19.1 (10.6)	2.0 to 55.5
Sugary foods and beverages^e^			15.8 (7.7)	2.0 to 53.0

^a^Data from DAGIS Survey (baseline) and DAGIS Next (follow-up).

^b^Low = high school or vocational diploma or less, medium = associate’s or bachelor’s degree, high = master’s, licentiate, or doctoral degree.

^c^As defined by Finnish growth reference standards.

^d^Fresh and cooked vegetables, fresh, frozen, canned fruits and fresh berries.

^e^Sweetened yogurts, quarks, puddings, cereals, berry soups, pastries and biscuits, chocolates, sweets, ice cream, and soft drinks and juices with added sugar.

### Baseline chronotype and social jetlag with follow-up diet and BMI *z*-score

Neither chronotype nor social jetlag were associated with dietary variables at follow-up (Table 3). Later chronotype and greater social jetlag in baseline were both associated with a higher follow-up BMI *z*-score in adjusted models. Every hour of a later chronotype during preschool age was associated with a 0.22 increase in BMI *z*-score in the eight-year follow-up (R^2^: 0.30 [95% CI: 0.20, 0.41]). For perspective, children in the earliest chronotype tertile had a mean (SD) of 01:36 (16 min), while children in the latest tertile had 03:00 (23 min). Likewise, every hour later, social jetlag in preschool-age was associated with a 0.33 increase in follow-up BMI *z*-score (R^2^: 0.30 [95% CI: 0.20, 0.41]). For additional perspective, children in the lowest social jetlag tertile had a mean (SD) of −3 (11) minutes, while those in the highest tertile experienced nearly an hour (53 (16) minutes) of social jetlag on average. Without adjusting for baseline BMI *z*-score, neither chronotype (β-est: 0.12 [95% CI: −0.10, 0.35], *p* = 0.27) nor social jetlag (β-est: 0.28 [95% CI: −0.06, 0.63], *p* = 0.10) predicted follow-up BMI *z*-score.

**Table 3:** Preschool-aged chronotype and social jetlag as predictors of consumption frequency of fruits and vegetables and sugary foods and beverages and BMI *z*-score eight years later in childhood in DAGIS^a^ study participants (*n* = 210).

	Model 1		Model 2	
	B-estimate (95% CI)	*p*-value	B-estimate (95% CI)	*p*-value
Chronotype				
Fruits and vegetables^b^	0.01 (−0.28, 0.29)	0.97	0.08 (−0.22, 0.37)	0.61
Sugary foods and beverages^c^	0.13 (−0.09, 0.35)	0.26	0.17 (−0.06, 0.40)	0.14
BMI *z*-score	**0.21 (0.02**, **0.40)**	**0.03**	**0.22 (0.01**, **0.42)**	**0.03**
Social jetlag				
Fruits and vegetables^b^	−0.16 (−0.61, 0.28)	0.46	−0.01 (−0.47, 0.45)	0.98
Sugary foods and beverages^c^	0.17 (−0.16, 0.50)	0.32	0.25 (−0.10, 0.60)	0.16
BMI *z*-score	**0.34 (0.05**, **0.63)**	**0.02**	**0.33 (0.02**, **0.65)**	**0.04**

Bolded values indicate statistical significance (*p* < 0.05).

^a^Data from DAGIS Survey (baseline) and DAGIS Next (follow-up).

^b^Fresh and cooked vegetables, fresh, frozen, canned fruits, and fresh berries.

^c^Sweetened yogurts, quarks, puddings, cereals, berry soups, pastries and biscuits, chocolates, sweets, ice cream, and soft drinks and juices with added sugar.

Model 1- Linear regression models with follow-up BMI z-score as outcome adjusted for baseline BMI z-score. Model 2- All linear regression models additionally adjusted for child’s baseline age, sex, parental highest education level, baseline sleep duration, and number of follow-up years in study.

Approximately two-thirds of the study population (*n* = 129) reported nurse measured follow-up height and weight. As a sensitivity analysis, analysis excluding home-measured height and weight still provided similar results for chronotype (β-est: 0.30 [95% CI: 0.06, 0.54], *p* = 0.02) and social jetlag (β-est: 0.60 [95% CI: 0.22, 0.97], *p* < 0.01) in fully adjusted models (models not presented in tables).

Analysis using BMI *z*-scores derived from International Obesity Task Force reference values yielded similar findings (Supplementary Table 4).

### Baseline chronotype and social jetlag with baseline BMI *z*-score

No cross-sectional associations were observed in baseline between chronotype or social jetlag and BMI *z*-score, even after adjustments for baseline covariates (β-est: −0.12 [95% CI: −0.36, 0.11], *p* = 0.30 and β-est: −0.06 [95% CI: −0.42, 0.31], *p* = 0.77, respectively) (models not presented in tables).

### Baseline chronotype and social jetlag with change in BMI *z*-score

Chronotype was associated with greater change in BMI *z*-score from baseline to follow-up in a crude model without any adjustments (β-est: 0.28 [95% CI: 0.05, 0.51], *p* = 0.02) and with the fully adjusted model (β-est: 0.24 [95% CI: 0.02, 0.45], *p* = 0.03). Similarly, social jetlag was associated with a greater change in BMI *z*-score in a crude model without any adjustment (β-est: 0.38 [95% CI: 0.04, 0.73], *p* = 0.03) and with the fully adjusted model (β-est: 0.36 [95% CI: 0.03, 0.69], *p* = 0.03). Chronotype and social jetlag both explained approximately 4% of the variance in change in BMI *z*-score in crude models and 27% for adjusted models (models not presented in tables).

## Discussion

This study examined the associations of chronotype and social jetlag in preschool-aged children with diet and weight outcomes during early adolescence in an eight-year follow-up study. Neither chronotype nor social jetlag in preschool age predicted the consumption frequencies of fruits and vegetables or sugary foods and drinks at follow-up. However, children who had later chronotype and greater social jetlag in preschool predicted a higher BMI z-score later in life when accounting for baseline BMI z-score.

Recent longitudinal evidence supports chronic social jetlag during adolescence to have long-term detrimental effects on weight outcomes. A combined cohort study with multiple repeated measures of sleep throughout an eleven-year span found that children with consistent or increasing social jetlag trajectories had a higher risk of obesity in young adulthood [45]. This is consistent with another study on school-aged children, which reported a modest association between an increase in social jetlag and higher BMI *z*-score after an average two-year follow-up [48]. In line with these longitudinal findings, our study suggests that these risks can potentially begin to develop at an even earlier stage in life.

Previous studies have generally found having a later chronotype to independently associate with increased weight status among older children [6], although studies on preschoolers have been less consistent. For instance, a sample of 116 preschoolers with six nights of actigraphy data observed no associations between sleep midpoint and adiposity in a one-year follow-up period [49]. Notably, this study is not directly comparable since the mean sleep midpoint across weekday and weekend nights is inherently different from chronotype, which reflects an adjusted sleep midpoint on days without morning obligations, like school. Having an affinity towards a later chronotype is a risk for circadian disruption only in the presence of misaligned biological and behavioral schedules, which is often inevitable for children attending school. This is further evidenced by researching lockdown periods during COVID-19 pandemic, where school-aged children adapted sleep timing more closely aligned to their biological preferences, and in doing so also reduced their social jetlag [50, 51]. Studying the mechanisms between these indicators of circadian misalignment risk and increased weight outcome risk in later life, particularly in the absence of less healthy dietary factors, falls outside of the scope of the current study. However, given the associations between having a later chronotype or greater social jetlag and sugary food and beverage consumption trended towards significance, it is possible that the sample size did not have sufficient power to detect true associations. Children exhibiting a later chronotype and greater social jetlag have been associated with less healthy dietary habits (e.g., lower vegetable consumption, higher intake of energy-dense foods) in cross-sectional settings, although the causal pathways remain unclear [21, 22, 32, 52]. Alternatively and regardless of diet, hindered glucose metabolism resulting directly from circadian misalignment could be an underlying mechanism [17]. However, more studies are needed to elucidate the direction of the effect and underlying mechanisms between sleep patterns and obesity risk throughout life.

This study has notable strengths, such as its follow-up design and comprehensive assessment of baseline measures including actigraphy-assessed sleep. Another strength is the investigation of the role of circadian misalignment risk indicators with diet and BMI z-score through a crucial stage of development between preschool age and early adolescence. However, as with any long follow-up period, there are potential changes in participant characteristics (e.g., changes in environment or family) that may have confounded the study findings. Likewise, unaccounted factors, such as genetic influence or pubertal development stage, may have additionally confounded the findings. In general, girls tend to exhibit maturation effects earlier. However, no interaction with sex was observed in the current study. The lack of cross-sectional associations between chronotype or social jetlag and BMI *z*-score at baseline suggests that the development of obesity in association to these potential circadian disruptors requires time.

One main limitation of this study is the small and selective sample. Only one-fourth of the baseline families participated in the follow-up study and those participating families had higher average parental education levels. Additionally, the prevalence of children with overweight was lower than national averages in Finland during follow-up [53]. Therefore, the study sample may not be representative of the general population. Moreover, since previous studies have suggested that populations with a higher risk of obesity or lower socioeconomic status may be more susceptible to the effects of sleep on diet and weight status [34, 54], it is possible that our findings have been attenuated. Participant-reported measures during the follow-up introduced potential bias, as did the use of different methods for collecting height and weight at the two study time points. However, a validation study among Finnish preadolescents found that the accuracy of home-measured anthropometrics was sufficient compared to researchers’ measurements [55]. Lastly, the potential risk of circadian disruption was evaluated only at baseline and from indicator variables such as phenotypical chronotype and social jetlag. Since young children have limited autonomy over their daily schedules, their sleep-related behaviors may reflect parental sleep habits, parenting styles, or home environments [56]. To better support childhood well-being, further research is needed to explore the connection between variables indicative of circadian health and modifiable family and household-level factors in younger children. Understanding these associations may provide valuable insights for improving circadian health and overall weight outcomes, especially for children most at risk.

Preschool-aged chronotype and social jetlag were able to predict later life BMI *z*-score, but not food consumption. Not surprisingly, the individual effect of chronotype and social jetlag in earlier childhood explained a small variation in preadolescent BMI *z*-score changes eight years later. Despite the understandably small effect sizes, the associations were both statistically and clinically significant, building on previous longitudinal findings [45, 48]. As the start of (pre)school may initiate change in sleep behavior for children, understanding the trajectories of behaviors throughout childhood is important in understanding the role of circadian health in diet and obesity. Researchers should consider collecting multiple repeated measures to assess trajectories of sleep traits, diet, and weight outcomes for future studies. Further research is warranted to develop intervention strategies that support children with obesity that also incorporate reducing the impact of sleep traits indicative of circadian misalignment.

## Supplementary information

Supplemental Material

## Data Availability

The datasets generated during and/or analyzed during the current study are not publicly available due ethical/privacy restrictions but are available from the PIs of the DAGIS Survey (ER) and DAGIS Next (RL) studies via corresponding author upon reasonable request. Imputation related analytic code is available on https://github.com/annaabdol/analysis-with-MICE.
