# Supplementary material for: Investigating preschool-aged chronotype and social jetlag as predictors of early adolescent diet and BMI z-score: an eight-year follow-up from the DAGIS study
Source: Int J Obes (Lond). 2024 Dec 19;49(5):793–800. doi: 10.1038/s41366-024-01702-4 (PMC12095071; doi:10.1038/s41366-024-01702-4)
Supplement: Supplementary file 1 — Supplemental Material [file 41366_2024_1702_MOESM1_ESM.docx]

**Supplementary Table 1.** Differences in baseline sociodemographic and behavioral characteristics of DAGIS^1^ participants excluded and included in the study population.

|  | Excluded study participants  (n=654) | Included study participants  (n=210) | p-value^2^ |
| --- | --- | --- | --- |
|  | mean (SD) or n (%) | mean (SD) or n (%) |  |
| Age (years) | 4.80 (0.90) | 4.69 (0.89) | 0.11 |
| Sex (female) | 311 (48%) | 100 (48%) | 1.0 |
| Parental education level^3^ |  |  | <0.001 |
| low | 180 (28%) | 21 (10%) |  |
| med | 273 (42%) | 82 (39%) |  |
| high | 197 (30%) | 106 (51%) |  |
|  |  |  |  |
| Weight-related variables^4^ |  |  |  |
| Overweight and obesity | 94 (14%) | 32 (15%) | 1.0 |
| BMI z-score, baseline | -0.02 (1.00) | -0.05 (0.97) | 0.69 |
|  |  |  |  |
| Sleep variables |  |  |  |
| Chronotype (hh:mm) | 02:18 (00:39) | 02:16 (00:40) | 0.90 |
| Social jetlag (min) | 26 (27) | 25 (26) | 0.62 |
| Sleep duration (hours) | 9.72 (0.52) | 9.73 (0.48) | 0.72 |

1. Data from DAGIS Survey (baseline). 2. p-values from independent t-test for continuous variables and chi-squared test for categorical variables. 3. Low = high school or vocational diploma or less, medium = associate’s or bachelor’s degree, high = master’s, licentiate, or doctoral degree. 4. As defined by Finnish growth reference standards.

**Supplementary Table 2.** Missing independent variable rates and means with and without imputation of missing data of DAGIS^1^ study participants (n=210)

| Independent variables | Missing (rate) | Pooled mean (SD) with imputations | Mean (SD) without imputations |
| --- | --- | --- | --- |
| Age (years) | 0 | - | - |
| follow-up years | 0 | - | - |
| Sex (female) | 0 | - | - |
| Parental education level^2^ | 1 (0.5%) |  |  |
| low |  | 21 (10%) | 21 (10%) |
| medium |  | 82 (39%) | 82 (39%) |
| high |  | 107 (51%) | 106 (51%) |
| BMI z-score, baseline | 7 (3%) | -0.06 (0.97) | -0.05 (0.97) |
| Chronotype (hh:mm) | 49 (23%) | 02:17 (00:39) | 02:16 (00:39) |
| Social jetlag (min) | 49 (23%) | 25 (26) | 25 (26) |
| Sleep duration (hh:mm) | 49 (23%) | 9.75 (0.49) | 9.73 (0.49) |

1. Data from DAGIS Survey (baseline) and DAGIS Next (follow-up). 2. Low = high school or vocational diploma or less, medium = associate’s or bachelor’s degree, high = master’s, licentiate, or doctoral degree.

**Supplementary Table 3.** Multivariable linear regression analyses of preschool aged chronotype and social jetlag as predictors of BMI z-score and food intake from later in childhood in DAGIS^1^ study participants without multiple imputation for missing data (n=159).

|  |  | Fully adjusted model | |
| --- | --- | --- | --- |
|  | n | estimate (95%CI) | p-value |
| Chronotype |  |  |  |
| Fruits and vegetables^2^ | 159 | 0.12 (-0.16, 0.40) | 0.41 |
| Sugary foods and beverages^3^ | 159 | 0.21 (-0.02, 0.45) | 0.08 |
| BMI z-score | 159 | **0.24 (0.02, 0.45)** | **0.03** |
| Social jetlag |  |  |  |
| Fruits and vegetables^2^ | 159 | 0.04 (-0.40, 0.47) | 0.87 |
| Sugary foods and beverages^3^ | 159 | 0.36 (-0.003, 0.73) | 0.05 |
| BMI z-score | 159 | **0.36 (0.03, 0.69)** | **0.03** |

1. Data from DAGIS Survey (baseline) and DAGIS Next (follow-up). 2. Fresh and cooked vegetables, fresh, frozen, canned fruits, and fresh berries. 3. Sweetened yogurts, quarks, puddings, cereals, berry soups, pastries and biscuits, chocolates, sweets, ice cream, and soft drinks and juices with added sugar. Fully adjusted model includes child’s baseline age, gender, parental highest education level at baseline, number of follow-up years, and baseline nighttime sleep duration. Models predicting BMI z-score are also adjusted for baseline BMI z-score.

**Supplementary Table 4.** Multivariable linear regression analyses of preschool-aged chronotype and social jetlag as predictors of consumption frequency and IOTF BMI z-score eight years later in childhood in DAGIS^1^ study participants (n=210).

|  | Model 1 |  | Model 2 |  |
| --- | --- | --- | --- | --- |
|  | B- estimate (95%CI) | p-value | B-estimate (95%CI) | p-value |
| Chronotype |  |  |  |  |
| Fruits and vegetables^2^ | -0.001 (-0.28, 0.28) | 0.99 | 0.07 (-0.22, 0.36) | 0.62 |
| Sugary foods and beverages^3^ | 0.12 (-0.10, 0.34) | 0.29 | 0.16 (-0.07, 0.39) | 0.17 |
| BMI z-score | **0.21 (0.02, 0.40)** | **0.03** | **0.23 (0.03, 0.43)** | **0.02** |
|  |  |  |  |  |
| Social jetlag |  |  |  |  |
| Fruits and vegetables^2^ | -0.17 (-0.59, 0.25) | 0.42 | -0.01 (-0.45, 0.42) | 0.95 |
| Sugary foods and beverages^3^ | 0.19 (-0.16, 0.54) | 0.29 | 0.27 (-0.10, 0.63) | 0.15 |
| BMI z-score | **0.35 (0.06, 0.64)** | **0.02** | **0.36 (0.05, 0.68)** | **0.02** |

IOTF: International Obesity Task Force. 1. Data from DAGIS Survey (baseline) and DAGIS Next (follow-up). 2. Fresh and cooked vegetables, fresh, frozen, canned fruits, and fresh berries. 3. Sweetened yogurts, quarks, puddings, cereals, berry soups, pastries and biscuits, chocolates, sweets, ice cream, and soft drinks and juices with added sugar. Model 1 – Models with follow-up BMI z-score as outcome adjusted for baseline BMI z-score. Model 2- All models additionally adjusted for child’s baseline age, sex, parental highest education level, baseline sleep duration, and number of follow-up years in study.
